# Crimean–Congo Hemorrhagic Fever in Children: Secondary Hemophagocytic Lymphohistiocytosis and Time-course of Biomarkers in a Single-Center Cohort (2019–25)

**DOI:** 10.1093/tropej/fmag048

**Published:** 2026-07-03

**Authors:** Aylin Irmak Kuruc, Kubra Aykac, Osman Oğuz Demir, Gamze Gürsoy, Tekin Aksu, Selin Aytaç, Ali Bulent Cengiz, Yasemin Ozsurekci

**Affiliations:** Department of Pediatric Infectious Diseases, Hacettepe University Faculty of Medicine, Ankara, Turkey; Department of Pediatric Infectious Diseases, Hacettepe University Faculty of Medicine, Ankara, Turkey; Department of Pediatric Infectious Diseases, Hacettepe University Faculty of Medicine, Ankara, Turkey; Department of Pediatric Hematology and Oncology Diseases, Hacettepe University Faculty of Medicine, Ankara, Turkey; Department of Pediatric Hematology and Oncology Diseases, Hacettepe University Faculty of Medicine, Ankara, Turkey; Department of Pediatric Hematology and Oncology Diseases, Hacettepe University Faculty of Medicine, Ankara, Turkey; Department of Pediatric Infectious Diseases, Hacettepe University Faculty of Medicine, Ankara, Turkey; Department of Pediatric Infectious Diseases, Hacettepe University Faculty of Medicine, Ankara, Turkey

## Abstract

Crimean–Congo hemorrhagic fever (CCHF) is a zoonotic viral infection that may present mildly in children but can progress to severe clinical states due to secondary hemophagocytic lymphohistiocytosis (HLH). We retrospectively analyzed the clinical, laboratory, and treatment data of children aged 0–18 years with polymerase chain reaction (PCR)-confirmed CCHF who were hospitalized at Hacettepe University, a large referral center for CCHF, between January 2019 and July 2025. Serial laboratory values (Days 0, 3, 7, and 10) and therapeutic interventions were evaluated. Twenty-two children (median age 13 years, 72% male) were included. The most frequent symptoms were fever (100%), bradycardia (59%), and conjunctival hyperemia (41%). HLH was diagnosed in 18 patients (82%). Ribavirin (91%), intravenous immunoglobulin (IVIG) (86%), and corticosteroids (68%) were administered. Platelet counts began to recover by Day 7, whereas ferritin declined gradually and normalized later. Aspartate aminotransferase (AST) and alanine aminotransferase (ALT) levels showed a progressive decrease. No patient required intensive care, and none died. Secondary HLH was highly prevalent among pediatric CCHF cases, yet outcomes were favorable with early antiviral and immunomodulatory therapy. Platelet recovery and delayed ferritin normalization may serve as practical biomarkers of disease resolution. Prospective multicenter studies with cytokine and viral-load profiling are warranted.

## Introduction

Crimean–Congo hemorrhagic fever (CCHF) is a tick-borne viral disease characterized by fever, hemorrhages, and multiorgan involvement. The principal vector is the *Hyalomma* tick, but the virus can also be transmitted through contact with blood or tissues of infected humans or animals, and occasionally by aerosol exposure [1]. Endemic areas include Africa, the Balkans, the Middle East, and Asia, including Turkey [2–4]. Recent reports indicate that changes in climate, travel, and host–vector dynamics are expanding the geographic distribution of CCHF, even into previously non-endemic regions of Europe [5, 6].

Typical clinical manifestations include fever, nausea, vomiting, diarrhea, headache, and myalgia, while conjunctival injection, bradycardia, and restlessness may also occur. The disease spectrum ranges from mild, self-limiting illness to severe hemorrhagic fever with high mortality [7, 8]. In children, disease severity is generally milder than in adults; however, a subset of pediatric patients can develop life-threatening complications such as secondary hemophagocytic lymphohistiocytosis (HLH) [4, 9, 10]. HLH is an acute, hyperinflammatory syndrome caused by excessive activation of macrophages and cytotoxic lymphocytes, leading to uncontrolled cytokine release, multiorgan failure, and potentially death. It may be primary (genetic) or secondary to triggers such as infections, malignancies, or autoimmune diseases [11–13]. The CCHF virus is one of the infectious agents known to precipitate secondary HLH through massive cytokine storm and immune dysregulation; however, pediatric data on this association remain extremely limited, with only rare reports available in the literature [14]. Although secondary HLH has been increasingly recognized as a severe complication of viral infections, its occurrence in the context of pediatric CCHF remains poorly characterized [15]. Current knowledge is largely derived from isolated case reports and small case series, with a lack of systematic data regarding its true frequency, clinical course, and laboratory evolution in children. This gap is particularly critical given that delayed recognition of HLH may significantly worsen outcomes in hyperinflammatory states such as CCHF [16, 17]. While HLH in adults with CCHF is increasingly recognized, systematic data in children are lacking, highlighting a significant gap in the pediatric literature [18].

With climate change, ecological shifts, increasing human–animal contact, and international travel, the geographic distribution of CCHF continues to expand, posing a growing global public health challenge. Early recognition of HLH, timely antiviral and immunomodulatory therapy, and close multidisciplinary monitoring are essential for improving survival in affected children [19].

CCHF is prevalent in low- and middle-income countries with limited access to advanced diagnostics, leading to under-recognition of pediatric secondary HLH [20]. Recognizing early laboratory trends and clinical predictors is crucial for enhancing outcomes in resource-limited settings [21]. Importantly, the identification of HLH in such settings often relies on widely available and relatively low-cost laboratory parameters, such as complete blood count and ferritin levels, rather than advanced immunological testing [13]. Given the scarcity of pediatric data and diagnostic challenges in low- and middle-income countries, this study aimed to evaluate the development of HLH, its clinical and laboratory features, and treatment responses in children with CCHF, thereby supporting earlier diagnosis and more feasible and scalable treatment strategies in such settings.

## Materials and methods

### Study design and setting

This retrospective study included pediatric patients (<18 years) who were diagnosed with CCHF and hospitalized at Hacettepe University İhsan Doğramacı Children’s Hospital between January 2019 and July 2025. CCHF diagnosis was confirmed by real-time reverse transcription–polymerase chain reaction (RT-PCR) at the Turkish Ministry of Health Reference Laboratory.

### Data collection

Clinical data were obtained from hospital medical records and included demographic information (age, sex, place of residence, admission date), clinical findings (fever, rash, headache, vomiting, diarrhea, weakness, epistaxis, bradycardia, conjunctival injection, etc.), laboratory parameters (complete blood count, ferritin, liver transaminases, and others), and treatment details [antiviral, corticosteroid, and intravenous immunoglobulin (IVIG) use, dose, and duration]. Serial laboratory measurements (Days 0, 3, 7, and 10) were collected whenever available. Due to variations in hospitalization duration (median 10.5 days; range 3–18 days), not all laboratory parameters were obtained at each predefined timepoint. Patients who were discharged before Day 10 received outpatient follow‑up for at least 7 days, and laboratory values from these visits were included in the analysis. Missing laboratory values were considered as missing data without imputation, following standard practices for retrospective observational cohorts.

### Ethical approval

Ethical approval was obtained from the Hacettepe University Faculty of Medicine Ethics Committee (approval no: SBA25-400). Written informed consent for clinical data and patient photographs was obtained from parents or legal guardians.

### Definitions

A probable case was defined as a patient meeting at least two of four clinical criteria (fever ≥38°C, fatigue, headache, generalized body aches or diarrhea; mucocutaneous bleeding; unexplained thrombocytopenia or leukopenia; or elevated transaminases) together with at least one epidemiologic criterion (tick exposure, contact with animal tissue/blood, residence in or travel to a rural endemic area, or close contact with a confirmed case). A confirmed case required laboratory confirmation by viral isolation, virus-specific IgM detection, ≥4-fold IgG titer rise, or detection of viral RNA by RT-PCR [22].

Index time (Day 0) was defined as the day of hospital admission, which served as the reference point for all temporal analyses unless otherwise specified. We acknowledge that the timing of admission may not coincide with the first symptom onset, which may create variability in assessments of disease course.

Secondary HLH was defined according to the HLH-2004 criteria, requiring ≥5 of 8 findings: fever, cytopenia (≥2 lineages), splenomegaly, hypofibrinogenemia and/or hypertriglyceridemia, hemophagocytosis in bone marrow/spleen/lymph nodes, hyperferritinemia, decreased NK-cell activity, or elevated soluble IL-2 receptor (sIL-2r) levels [12, 13].

Bradycardia was defined as a heart rate below the lower limit of normal for age [23].

The Ribavirin protocol begins with an initial dose of 30 mg/kg/dose on the first day, followed by 15 mg/kg/dose for 4 days, and then 7 mg/kg/dose for 6 days if clinically necessary such as in case of resistant thrombocytopenia [24]. IVIG was administered to patients at a dose of 2 g/kg within 12 h of admission, either as a 48-hour infusion or at a dose of 400–500 mg/kg/day over 4–5 days. Steroid treatment options include methylprednisolone (1–10 mg/kg/day) or dexamethasone (10 mg/m^2^/day) as initial therapy.

The initiation of corticosteroids and/or IVIG was guided by the presence of secondary HLH or strong clinical suspicion of hyperinflammation. Treatment decisions were based on a combination of clinical and laboratory findings, including persistent fever, cytopenia affecting ≥2 cell lines, markedly elevated ferritin levels, and evidence of clinical deterioration (e.g. worsening cytopenia or organ dysfunction) [13]. In patients who did not fully meet HLH-2004 criteria, therapy was initiated at the discretion of the multidisciplinary team when hyperinflammatory features were considered significant.

### Statistical analysis

Data were analyzed using IBM SPSS Statistics version 25.0 and GraphPad Prism version 10. Continuous variables were summarized as median (minimum–maximum) and categorical variables as number (percentage) for baseline descriptive analyses. For longitudinal evaluation of laboratory parameters measured at predefined time points, mean values with standard deviation (mean ± SD) were used to better illustrate temporal trends across repeated measurements.

As all patients formed a single cohort, no between-group comparisons were made. Instead, descriptive and longitudinal analyses were performed to evaluate temporal changes in laboratory parameters [absolute lymphocyte count, platelet count, ferritin, aspartate aminotransferase (AST), and alanine aminotransferase (ALT)] measured on Days 0, 3, 7, and 10.

## Results

**Figure 1:**
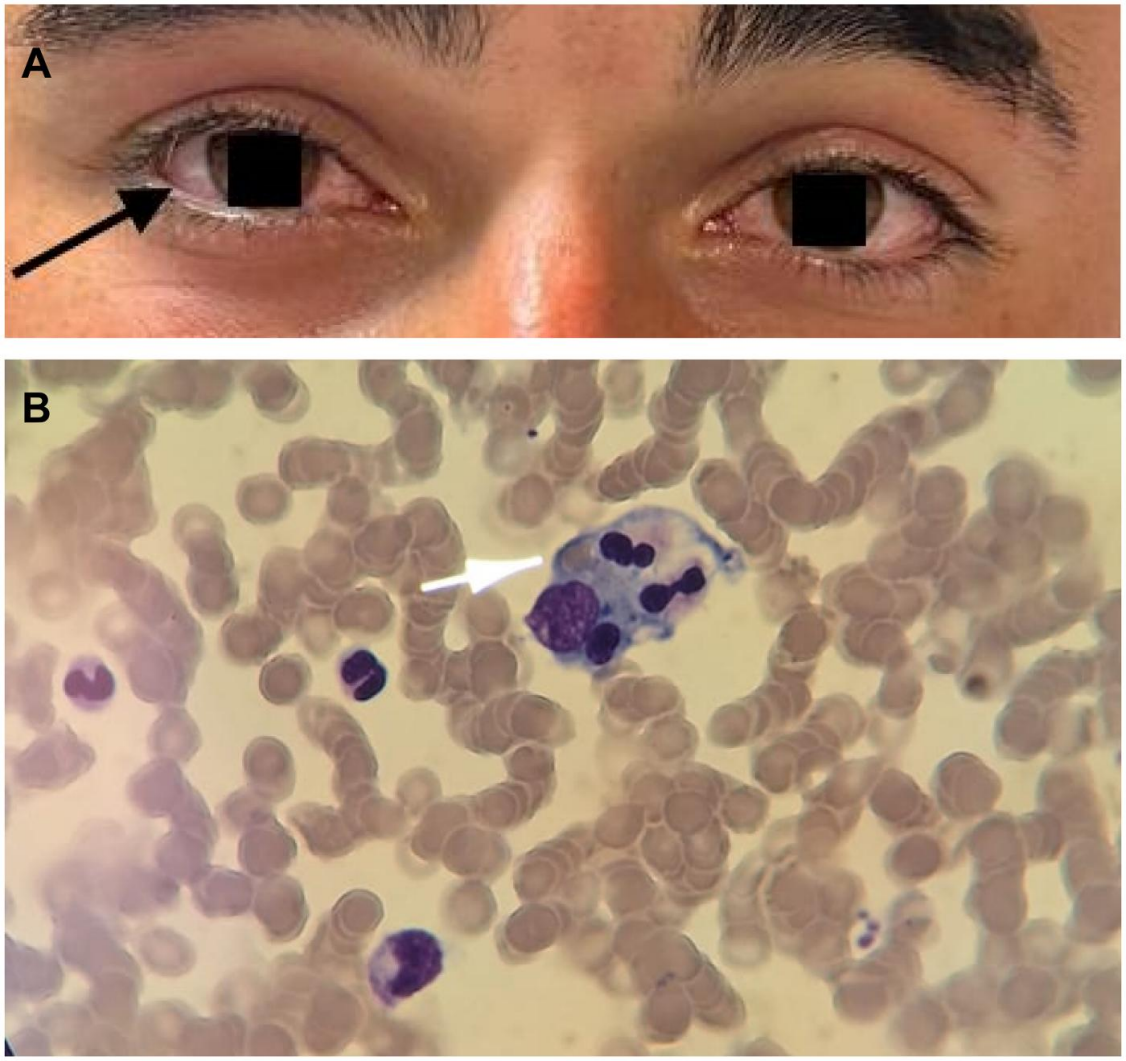
(A) Conjunctival injection in a child with Crimean–Congo hemorrhagic fever (CCHF) who developed secondary hemophagocytic lymphohistiocytosis (HLH). Patient photograph was taken and published with family and patient consent. (B) Hemophagocytic cells evaluated in the patient’s bone marrow smear. The smear demonstrates activated macrophages engulfing hematopoietic elements, consistent with hemophagocytic activity observed in secondary HLH.

A total of 22 children with confirmed CCHF were included in the study. Sixteen (72.2%) patients were male, and the median age was 13 years (min–max, 2–17 years). Forty-one percent of the patients resided in the Central Anatolia region, and considering the seasonal distribution, almost all cases were seen during the spring and summer months. All patients presented with fever (100%). The most frequent accompanying findings were bradycardia (59.1%) and conjunctival hyperemia (40.9%) (Fig. 1A). Other symptoms included nausea/vomiting (31.8%), fatigue (27.3%), and abdominal pain (13.6%) (Table 1).

**Table 1:** Clinical and demographic data of patients diagnosed with CCHF.

Age, years, median (min–max)	13 (2–17)
Sex, male *n* (%)	16 (72)
Clinical findings, *n* (%)	
Fever	22 (100)
Headache	4 (18)
Nausea/vomiting	7 (32)
Weakness	6 (27)
Restlessness	1 (4.5)
Diarrhea	4 (18)
Abdominal pain	4 (18)
Myalgia	1 (4.5)
Cough	1 (4.5)
Nosebleeds	1 (4.5)
Conjunctival injection	9 (41)
Maculopapular rash	4 (18.)
Bradycardia	13 (59.)
HLH development, *n* (%)	18 (82)
Treatment	
Ribavirin *n* (%)	20 (91)
IVIG	19 (87)
Steroid *n* (%)	15 (68)
Methylprednisolone	6 (27)
Dexamethasone	9 (41)
Treatment duration (day, min–max)	9 (5–13)
LOS, day, median (min–max)	10.5 (3–18)
Mortality	0

HLH, hemophagocytic lymphohistiocytosis; IVIG, intravenous immunoglobulin; LOS, length of hospital stay.

Among pediatric patients diagnosed with CCHF, the frequency of secondary HLH increased notably across successive time periods. Secondary HLH was identified in 1 of 3 patients (33.3%) during the pre-pandemic period (2019–20), in 11 of 12 patients (91.7%) during the COVID-19 pandemic years (2020–22), and in 6 of 7 patients (85.7%) in the post-pandemic period (2022–25).

**Figure 2:**
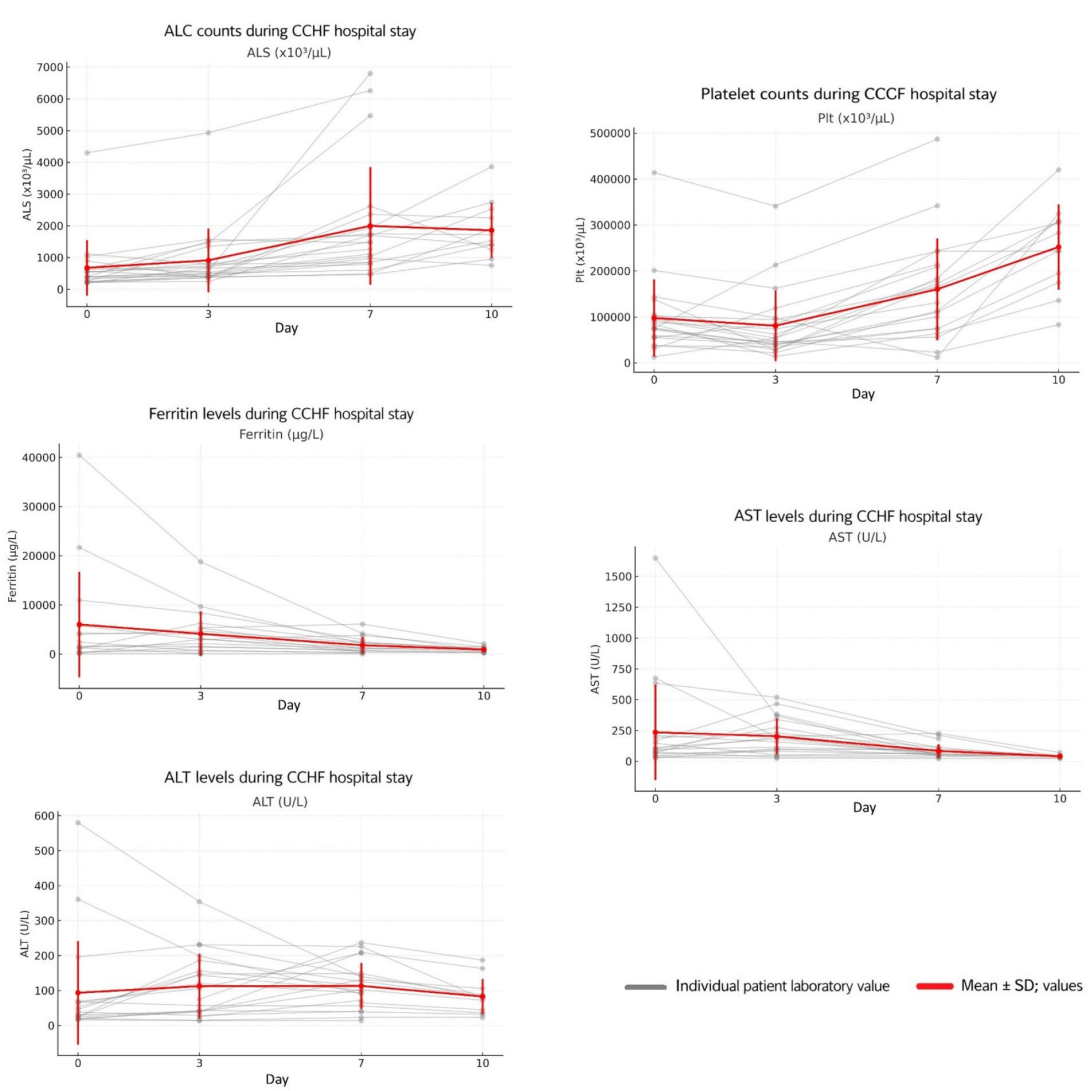
Longitudinal changes in laboratory parameters during hospitalization in pediatric patients with CCHF. Absolute lymphocyte count (ALC; ×10³/µl), platelet count (Plt; ×10³/µl), ferritin (µg/l), aspartate aminotransferase (AST; U/l), and alanine aminotransferase (ALT; U/l) levels are shown at Days 0, 3, 7, and 10. Individual patient values are shown as separate lines, while mean values with standard deviation are indicated. Gray lines represent individual patient values, while red lines indicate mean values with standard deviation (mean ± SD).

Laboratory parameters of the patients were evaluated longitudinally on a daily basis. At admission (Day 0), platelet counts were markedly decreased (mean 97 × 10³/µl ± 83 × 10³) and showed a significant upward trend over time (Friedman *P* < .05), reaching 252 × 10³/µl ± 92 × 10³ on Day 10. Ferritin levels were markedly elevated at baseline (mean 6020 µg/l ± 10 736) and decreased gradually but remained above normal limits on Day 10 (mean 901 µg/l ± 566). AST levels declined from 236 ± 388 U/l at admission to 41 ± 12 U/l by Day 10. Similarly, ALT decreased from 94 ± 148 U/l to 83 ± 50 U/l, while absolute lymphocyte counts rose from 675 ± 872 × 10³/µl on Day 0 to 1998 ± 1852 × 10³/µl on Day 7 and stabilized thereafter (Fig. 2).

Eighteen patients (81.8%) fulfilled the diagnostic criteria for secondary HLH. Among them, bone marrow aspiration was performed in 11 patients, and hemophagocytosis was identified in all (Fig. 1B).

The median hospital stay was 10.5 days, (range 3–18). Twenty patients (90.9%) received ribavirin, 19 (86.4%) received IVIG, and 15 (68.2%) received corticosteroids. Among patients with HLH (*n* = 18), triple therapy with ribavirin, IVIG, and corticosteroids was administered in 15 cases, while two received ribavirin + IVIG and one received ribavirin + steroid. In contrast, among patients without HLH (*n* = 4), two received ribavirin + IVIG and two did not receive any specific treatment. None of the patients required intensive care, mechanical ventilation, or inotropic support. Fourteen patients received fresh-frozen plasma and 10 required platelet transfusion. No treatment-related adverse effects or deaths occurred; all patients were discharged in full recovery.

## Discussion

CCHF is a zoonotic infection whose incidence has increased in recent years due to climate change, altered tick ecology, and expanding human–animal interaction and international travel [25, 26].

CCHF disproportionately affects low‑ and middle‑income countries, where diagnostic capacity, laboratory infrastructure, and access to advanced immunological testing remain limited. Recent analyses from Africa and Asia highlight that under‑recognition of severe complications such as HLH is partly driven by these resource constraints, resulting in delayed diagnosis and suboptimal clinical outcomes [20, 21, 27]. In such settings, reliance on simple, widely available laboratory markers becomes crucial. Therefore, improving awareness of HLH, as a potential complication of CCHF and identifying early predictors are particularly important for clinicians working in resource-limited environments [13].

Importantly, our study was conducted in a middle-income country with healthcare conditions comparable to many CCHF-endemic regions, reflecting real-world clinical practice rather than highly specialized settings. The laboratory parameters highlighted in our analysis, including platelet count, ferritin, and transaminases, are routinely available in most secondary and tertiary care centers and do not require advanced or costly testing.

CCHF is also associated with a considerable healthcare burden, including hospitalization-related costs [28]. From a treatment perspective, commonly used interventions such as ribavirin and corticosteroids are generally accessible and relatively affordable in many endemic regions, whereas access to IVIG may be limited due to cost and availability, particularly in low- and middle-income countries [29, 30]. In this context, early recognition of HLH based on clinical and basic laboratory findings may allow timely initiation of more accessible therapies, particularly corticosteroids, even in resource-constrained settings. Taken together, these features enhance the feasibility, reproducibility, and generalizability of our findings, supporting their applicability across low- and middle-income countries where CCHF remains a significant public health concern.

Although CCHF usually presents with a milder clinical course in children than in adults, the development of secondary HLH may dramatically worsen the disease outcome [14, 19, 31]. HLH represents a severe hyperinflammatory syndrome triggered by excessive cytokine release, and its recognition in the context of pediatric CCHF remains clinically challenging yet critical for early intervention [14, 32]. In our cohort, secondary HLH developed in 81.8% of children with CCHF. The true frequency of HLH in CCHF remains uncertain, as most of the existing evidence is derived from isolated case reports or small series. In one early Turkish study involving 14 adult patients with CCHF, reactive hemophagocytosis was detected in 7 (50%) cases, suggesting that HLH may contribute to disease pathogenesis rather than being a rare occurrence [33]. Beyond such limited observations, pediatric data remain scarce, with most publications describing only single cases. The high proportion observed in our study may therefore reflect both referral bias, since our center predominantly receives severe cases from endemic region. Remarkably, despite the high proportion of severe cases and HLH in our cohort, all patients survived and all were discharged in full recovery. This outcome contrasts with the mortality rates of 5–30% reported in the general CCHF literature [3, 7, 19] and underscores the potential impact of early antiviral, IVIG, and corticosteroid therapy combined with meticulous multidisciplinary management [24]. Moreover, our findings emphasize the importance of heightened clinical awareness for HLH as a potential complication of CCHF, as early recognition and prompt treatment appear to be crucial for improving outcomes.

Serial laboratory evaluation demonstrated dynamic changes in hematologic and biochemical parameters over the disease course. Platelet counts began to recover significantly by Day 7, indicating early hematologic improvement, while ferritin levels declined more slowly and remained elevated at Day 10, suggesting persistent inflammatory activity. This temporal pattern supports the interpretation that hematologic recovery precedes the resolution of hyperinflammation [14]. These trends, particularly the early rise in platelet count and delayed decline in ferritin, highlight the potential role of these parameters as practical biomarkers for monitoring disease resolution and inflammatory burden in pediatric CCHF.

Severe CCHF is characterized by high viral load and a strong pro-inflammatory cytokine response (TNF-α, IL-6, IL-8, etc.), which contributes to systemic endothelial injury and multi-organ damage, including the liver [34]. CCHFV infects hepatocytes and sinusoidal endothelial cells, inducing ER-stress–mediated apoptosis and focal necrosis. Simultaneously, high viremia drives a strong cytokine response and endothelial dysfunction, causing microvascular leakage and ischemia [4].

A meta-analysis of CCHF-induced liver injury found that abnormal liver function tests (LFT) are very common and that liver injury is significantly associated with increased mortality and need for intensive care [35]. In our clinical cohort, we observed that patients who showed overall improvement also demonstrated a rapid normalization of LFTs. Fortunately, none of our patients experienced fatal outcomes.

All patients in this cohort received comprehensive supportive care, and most were treated with a combination of antiviral and immunomodulatory agents. Ribavirin was administered to 91% of patients, consistent with national treatment recommendations, and its early use may have contributed to viral suppression and improved outcomes. IVIG and corticosteroids were used in 86% and 68% of cases, respectively, primarily to mitigate hyperinflammation associated with HLH.

Although causality cannot be inferred from this retrospective design, the combination of early antiviral therapy and timely immunomodulation appears to have played a critical role in preventing mortality in our cohort. Previous studies have similarly reported that prompt ribavirin administration can reduce viral replication and fatality rates [24, 36], while adjunctive therapies targeting cytokine storm, such as IVIG and corticosteroids, may help attenuate the hyperinflammatory phase of secondary HLH. Recent international priorities for CCHF research also emphasize the urgent need to characterize host immune responses and develop targeted immunomodulatory interventions [37], supporting the relevance of our findings to global disease management strategies. Multidisciplinary monitoring and individualized therapy likely contributed to the favorable clinical outcomes observed.

The marked increase in secondary HLH among pediatric patients with CCHF across successive periods may reflect broader post-pandemic alterations in host immune responses. In our cohort, the proportion of children developing secondary HLH rose over 85% during and after the COVID-19 era. Similar trends have been documented for several viral infections including respiratory syncytial virus, influenza, adenovirus, and enterovirus where disease severity increased after the relaxation of pandemic mitigation measures. Proposed mechanisms include “immune debt” caused by reduced viral exposures during lockdowns, subsequent immune naivety in younger age groups, and post-pandemic immune dysregulation characterized by exaggerated inflammatory responses [38, 39]. CHF is a viral infection in which high cytokine output and dysregulated host immunity are central to pathogenesis [40]; therefore, a heightened inflammatory milieu in the post-pandemic pediatric population could plausibly lower the threshold for triggering secondary HLH. Although causality cannot be definitively established, our findings are consistent with the hypothesis that post-pandemic immune shifts may potentiate hyperinflammatory complications in susceptible infections such as CCHF. Further multicenter studies incorporating immunologic profiling and viral kinetics are warranted to clarify these relationships.

### Strengths and limitations

This study has several strengths. First, it provides one of the few systematic evaluations of secondary HLH in pediatric CCHF, addressing an important gap in the literature. Second, the longitudinal analysis of laboratory parameters at predefined time points allowed a detailed assessment of the temporal evolution of hematologic and inflammatory markers, offering clinically relevant insights into disease monitoring. Third, the study reflects real-world clinical practice in a middle-income, CCHF-endemic setting, enhancing the applicability and generalizability of the findings to similar resource-limited regions.

This study has several limitations. First, its retrospective, single-center design limits the generalizability of the observed increase in secondary HLH across time periods, particularly in the context of potential post-pandemic shifts in immune responses. Second, the relatively small sample size reduced the statistical power and prevented robust multivariable modeling to disentangle the contribution of clinical, virological, and temporal factors to HLH development. Because of the small sample size and the individualized nature of treatment allocation, comparative analyses between treatment groups could not be performed. Larger multicenter cohorts are needed to evaluate potential differences in treatment response. Third, viral load measurements, cytokine profiling, and immune phenotyping were not available, restricting our ability to explore mechanistic links between CCHFV infection, post-pandemic immune changes, and hyperinflammatory complications. Finally, as no parallel population-level data on other viral infections were collected, we cannot determine whether the increased HLH frequency reflects a broader post-pandemic phenomenon or characteristics unique to our cohort. Despite these limitations, our study provides important real-world insight into the evolving clinical spectrum, laboratory trajectories, and outcomes of pediatric CCHF, highlighting the need for larger multicenter studies incorporating detailed immunologic and virologic assessments.

## Conclusion

In conclusion, secondary HLH was highly prevalent among children with CCHF in our cohort. Despite the high frequency of HLH, all patients recovered without mortality following antiviral or immunomodulatory therapy. Platelet counts showed early recovery, whereas ferritin levels declined more gradually during follow-up. These findings highlight the importance of early recognition and close monitoring of hyperinflammatory features in pediatric CCHF.

## Data Availability

The data generated during the current study are available from the corresponding authors on a reasonable request.

## Use of large language models (LLMs)

AI-assisted copy editing was used to improve the readability, grammar, and style of the manuscript text. All AI-assisted content was carefully reviewed, edited, and approved by the authors to ensure accuracy and accountability.
